# From Automated ECG Interpretation to Multimodal Cardiovascular Intelligence: The Evolution of Artificial Intelligence in Cardiovascular Medicine

**DOI:** 10.3390/medsci14040434

**Published:** 2026-07-25

**Authors:** Lavinia Rech

**Affiliations:** Independent Researcher, 80993 Munich, Germany; lavinia.rech@cardioscience-and-more.com

**Keywords:** artificial intelligence, cardiovascular disease, digital health, cardiovascular imaging, multimodal artificial intelligence, clinical decision support

## Abstract

Artificial intelligence (AI) is rapidly transforming cardiovascular medicine, driven by the increasing availability of large-scale clinical data and advances in machine learning. Early computational applications in cardiology were primarily limited to rule-based electrocardiogram interpretation systems. Over time, these approaches have evolved into sophisticated deep learning models capable of analysing complex cardiovascular signals and imaging data. In parallel with the broader development of digital health technologies, including wearable devices, electronic health records, and remote monitoring systems, AI applications have expanded across multiple domains of cardiovascular care. These now include electrocardiographic (ECG) and electrophysiological analysis, cardiovascular imaging, surgical planning, and multimodal risk prediction. More recently, multimodal AI models have emerged that integrate heterogeneous data sources such as imaging, physiological signals, clinical records, and genomic information, enabling more comprehensive characterisation of cardiovascular disease. Beyond diagnostic applications, AI is increasingly influencing system-level aspects of cardiovascular medicine, including clinical decision support, workflow optimisation, medical education, and clinical trial design. This narrative review traces the historical and clinical evolution of artificial intelligence in cardiovascular medicine from early automated ECG interpretation systems to contemporary multimodal and system-level applications. It highlights key technological developments, current clinical applications, translational challenges, and the emerging role of AI within digital cardiovascular health ecosystems, with particular emphasis on early disease detection, risk stratification, prognostic modelling, and personalised cardiovascular care.

## 1. Introduction

Cardiovascular diseases remain the leading cause of morbidity and mortality worldwide [1], and their diagnosis and management rely on increasingly complex and diverse data, from electrocardiographic signals and multimodal imaging to electronic health records, genomic information, and wearable-derived physiological measurements [2]. Interpreting this wide range of data at scale has prompted the integration of computational methods into cardiovascular care, evolving from early rule-based algorithms for ECG interpretation to the advanced deep learning frameworks available today.

This development has not occurred in isolation. It is closely linked to the wider transformation of cardiovascular medicine through digital health technologies, including wearable sensors, remote monitoring platforms, and interoperable health data systems. These innovations both enable and demand more intelligent analytical tools. While existing reviews have often focused on individual modalities, specific disease entities, or technical aspects of cardiovascular AI, including modality-centred imaging applications, fewer have traced the broader historical and clinical evolution of AI across the full spectrum of cardiovascular medicine. Such an integrative perspective is increasingly important because the clinical value of AI is shifting from isolated diagnostic automation towards multimodal risk stratification, early disease detection, longitudinal monitoring, and system-level decision support within digital cardiovascular health ecosystems.

Studies were selected for inclusion based on their clinical relevance, methodological influence, and contribution to illustrating the historical development or future implementation of AI in cardiovascular medicine. This review synthesises clinically significant and representative literature on cardiovascular artificial intelligence (AI), with an emphasis on landmark studies, translational relevance, and emerging implementation challenges. Relevant literature was identified primarily through PubMed using topic-specific searches across the major thematic domains covered in this review during the initial development of the manuscript and updated until April 2026. Additional landmark publications were identified through citation tracking of key reviews and seminal articles, supplemented where appropriate by broader literature searches. Priority was given to peer-reviewed, English-language original studies, major reviews, consensus documents, and publications addressing clinical translation or implementation. Non-peer-reviewed sources were generally excluded, except when primary regulatory or policy documents were required. When findings differed across publications, they were interpreted in relation to study design, population, data source, validation setting, and translational context.

This narrative review therefore provides an integrative historical overview of the evolution of AI in cardiovascular medicine, from automated ECG interpretation to modern multimodal AI systems. By following this trajectory from single-modality interpretation to integrated cardiovascular intelligence, the review emphasises how AI may support earlier recognition of subclinical disease, more refined prognostic assessment, and more personalised prevention and treatment strategies. It highlights key technological milestones, current clinical uses, their impact on cardiovascular diagnostics and the emerging role of AI within digital cardiovascular health ecosystems. Across these domains, the evidence is also considered through a cross-cutting translational lens, distinguishing technical development and validation from prospective evaluation, regulatory authorisation, routine clinical implementation and demonstrated clinical benefit.

## 2. Evolution of Artificial Intelligence in Cardiovascular Medicine

**Figure 1:**
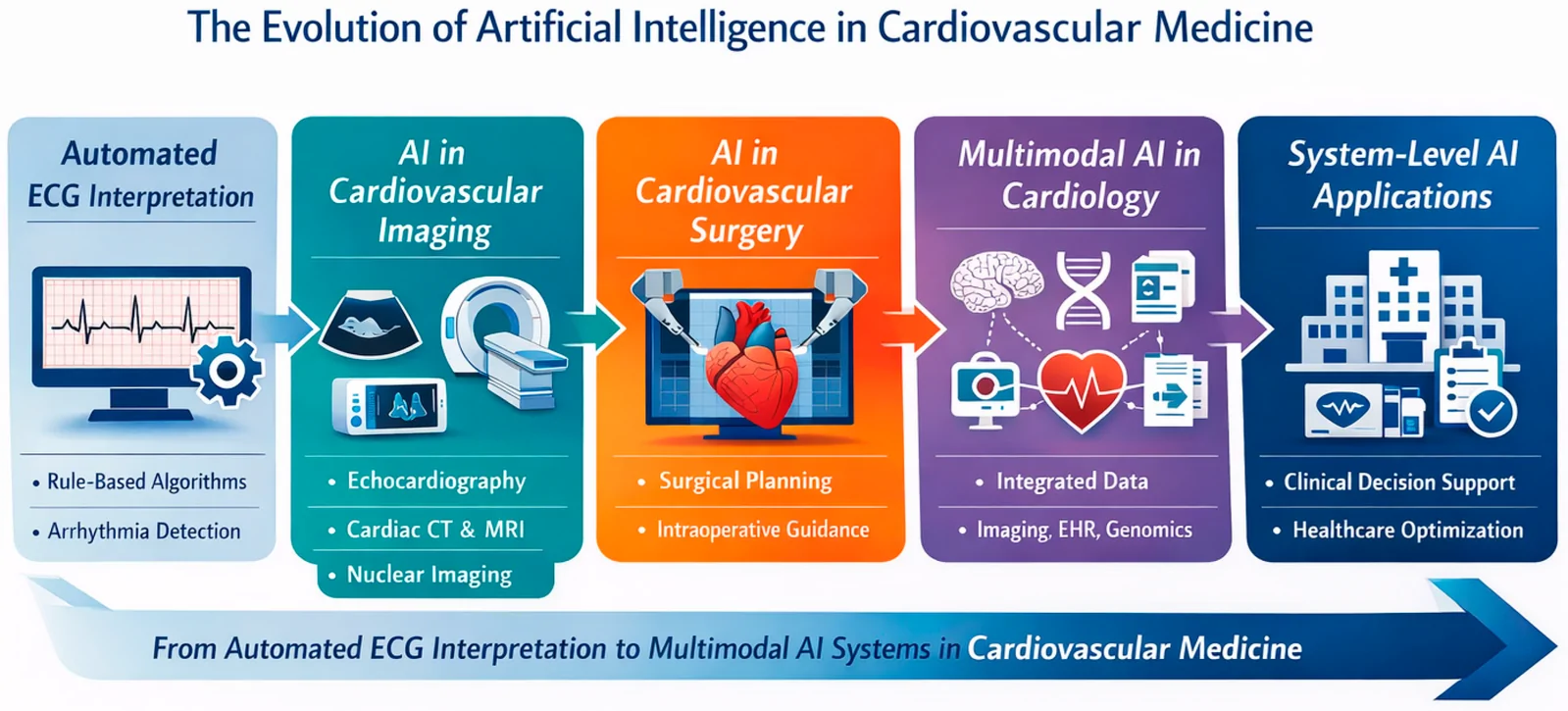
Historical trajectory from automated ECG interpretation to multimodal and system-level artificial intelligence in cardiovascular medicine.

The development of AI in cardiovascular medicine has advanced steadily across different diagnostic and clinical areas. Early applications focused on automated analysis of physiological signals, before expanding to include sophisticated imaging techniques and computational decision-support tools. Progress in machine learning and deep learning has enabled more complex methods, from automated image analysis to multimodal models that integrate imaging, physiological, and clinical data. The following sections outline the progression of AI in key areas of cardiovascular diagnostics and clinical practice. The overall development from early rule-based ECG interpretation to modern multimodal AI frameworks is summarised in Figure 1.

### 2.1. Artificial Intelligence in Electrocardiography and Cardiac Electrophysiology

#### 2.1.1. Artificial Intelligence in Electrocardiography

Automated electrocardiogram (ECG) interpretation is one of the earliest computer-assisted applications in cardiovascular medicine [3]. Early systems relied on rule-based algorithms to assist clinicians in recognising basic electrical patterns, such as arrhythmias, conduction abnormalities, and ventricular hypertrophy. Although useful as supportive tools in routine clinical practice, these early approaches were limited by rigid decision rules and reduced adaptability to complex signal patterns, which sometimes resulted in clinically relevant misinterpretations.

Current AI-ECG applications can be broadly grouped into three intended use cases: rhythm detection and monitoring, prediction of structural or functional cardiovascular disease, and physiological or extracardiac phenotyping. These use cases differ substantially in their target populations, validation requirements, downstream consequences, and readiness for clinical implementation.

Over the past decade, advances in AI, especially deep learning convolutional neural networks (CNNs), combined with the availability of large annotated ECG datasets, have significantly enhanced automated ECG analysis [4]. Originally developed for arrhythmia detection, modern AI-based ECG models can now recognise a wide range of rhythm disorders with diagnostic accuracy nearing that of expert cardiologists. For instance, a deep neural network trained on over 90,000 single-lead ECG recordings achieved cardiologist-level performance in classifying multiple arrhythmias [5]. Similarly, large-scale deep learning approaches have shown the ability to detect atrial fibrillation even when the ECG is recorded in normal sinus rhythm, indicating that AI may identify subtle electrical signatures that are invisible to traditional interpretation [6,7].

Beyond traditional rhythm classification, AI-enabled ECG analysis can extract much more complex phenotypic information from electrical signals. Deep learning models trained on large ECG repositories have demonstrated the ability to detect structural and functional cardiac abnormalities, including hypertrophic cardiomyopathy, left ventricular systolic dysfunction, and even cardiac allograft rejection, conditions that are not directly visible on conventional ECG interpretation [8]. In particular, AI-based ECG screening has shown promise for identifying asymptomatic or “silent” atrial fibrillation and left ventricular dysfunction, supporting earlier detection and enabling scalable population-level screening strategies through wearable devices and remote monitoring platforms [9,10]. Some AI-enabled ECG applications have already moved beyond purely experimental settings, particularly in rhythm monitoring, where algorithm-supported wearable and mobile ECG systems have received regulatory clearance and are increasingly used for atrial fibrillation detection and remote rhythm surveillance [8,11].

More recently, research has suggested that AI-based ECG analysis may function as a broader physiological biomarker beyond cardiovascular disease. Deep learning models have demonstrated the potential to detect extracardiac physiological disturbances, such as electrolyte abnormalities, including hyper- and hypokalaemia [12], and even neurological disorders such as Parkinson’s disease [13]. These findings illustrate how advanced AI approaches can uncover subtle signal features embedded within ECG waveforms, highlighting the expanding role of ECG-based AI as a non-invasive tool for integrated physiological monitoring.

However, the clinical maturity of AI-ECG applications varies substantially across indications. While rhythm detection and selected monitoring applications are already entering routine care, many broader applications, including prediction of left ventricular dysfunction, electrolyte abnormalities, allograft rejection, or extracardiac disease, remain largely investigational. Most models have been developed and validated retrospectively, and their performance may decline in external populations with different ECG acquisition systems, comorbidity profiles, disease prevalence, or clinical workflows [4,8]. In population-level screening, false-positive results, downstream testing, patient anxiety, and uncertain effects on clinical outcomes must therefore be considered before widespread implementation. Future ECG-based AI systems will require robust external validation, prospective evaluation, and clear integration into clinical decision pathways to demonstrate not only diagnostic performance but also clinical utility. Evaluation should therefore be use-case-specific and consider the intended population, disease prevalence, calibration, consequences of false positives, transportability, and incremental value over conventional ECG interpretation or existing screening pathways.

#### 2.1.2. Artificial Intelligence in Cardiac Electrophysiology

Artificial intelligence applications in cardiac electrophysiology extend beyond automated interpretation of surface electrocardiograms and increasingly encompass intracardiac signal analysis, electroanatomical mapping, catheter ablation, implantable cardiac devices, and prediction of treatment outcomes. Machine-learning and deep-learning methods can process heterogeneous datasets comprising electrocardiographic and intracardiac signals, imaging findings, clinical variables, device recordings, and procedural information. These approaches are being investigated across the electrophysiological care pathway, from arrhythmia detection and risk stratification to procedural planning, treatment selection, and longitudinal monitoring [14,15,16,17].

A major area of development is the analysis of intracardiac electrograms and electroanatomical mapping data. Conventional interpretation of intracardiac signals relies heavily on operator expertise and can be affected by signal complexity, noise, and variability across mapping systems. AI-based methods may support automated recognition of abnormal conduction patterns, electrogram fractionation, arrhythmogenic substrates, and potential sites of arrhythmia origin. When integrated with three-dimensional electroanatomical maps or cardiac imaging, these approaches may improve substrate characterisation and assist in identifying potential ablation targets. Computational modelling and machine learning are also being explored for non-invasive or pre-procedural localisation of arrhythmogenic regions, potentially enabling more individualised procedural strategies [14,15,16,17].

AI may further support catheter ablation through pre-procedural risk assessment, intra-procedural decision support, and post-procedural outcome prediction. Models that integrate clinical characteristics, imaging, surface or intracardiac electrophysiological data, and procedural variables have been developed to estimate the risk of recurrence after ablation for atrial fibrillation or ventricular arrhythmias. Such models may eventually assist with patient selection, counselling, procedural planning, and risk-adapted follow-up. During procedures, AI-supported analysis may aid in catheter navigation, interpretation of mapping, lesion assessment, and procedural workflow recognition. However, most of these applications remain investigational, and evidence that AI-guided strategies improve procedural efficiency, freedom from recurrent arrhythmia, safety, or patient-relevant outcomes beyond established electrophysiological approaches remains limited [14,15,16,17].

Implantable cardiac devices also generate longitudinal intracardiac electrophysiological data that may support AI-based risk stratification and device management. Machine-learning models have been investigated for analysing intracardiac electrograms, arrhythmia burden, and device-derived physiological parameters, aiming to improve the detection of atrial and ventricular arrhythmias, identify patients at increased risk of recurrence or clinical deterioration, and support more individualised follow-up. However, the clinical value of such models depends on adequate calibration, manageable false-positive rates, compatibility across device platforms, and evidence that predictive alerts lead to meaningful changes in management or outcomes rather than adding to the monitoring burden [14,15,16].

Despite substantial technical progress, translation into routine electrophysiological practice remains uneven. Many models have been developed retrospectively using data from individual centres, selected patient populations, or specific acquisition and mapping platforms. Their transportability may therefore be limited by differences in signal quality, hardware, procedural techniques, arrhythmia prevalence, annotation practices, and clinical workflows. Prospective multicentre evaluation should assess not only discrimination or classification accuracy but also calibration, clinical decision-making, procedural efficiency, consequences of false positives, safety, and patient outcomes. Explainability, interoperability, regulatory oversight, and post-deployment surveillance will also be important, particularly for systems that provide real-time procedural recommendations or continuously analyse implantable-device data. Accordingly, AI in electrophysiology currently appears most appropriate as a clinician-support technology, with its incremental value over established mapping, monitoring, and risk-stratification strategies requiring further demonstration [14,15,16,17].

### 2.2. Artificial Intelligence in Cardiovascular Imaging

Cardiovascular imaging is one of the fastest-growing areas of AI in medicine. Early computational methods used rule-based image analysis techniques, often called computer-aided diagnosis (CAD), which were initially created for radiographic imaging and later modified for cardiovascular uses like coronary angiography [18,19]. With the rise of machine learning and deep learning, imaging analysis has transitioned from handcrafted feature extraction to data-driven pattern recognition. Nowadays, AI facilitates automated image interpretation, quantitative analysis, and risk prediction across various cardiovascular imaging techniques, including echocardiography, nuclear imaging, vascular ultrasound, cardiac magnetic resonance, and computed tomography [2].

#### 2.2.1. Nuclear Imaging (SPECT and PET)

Nuclear cardiology, especially single-photon emission computed tomography (SPECT) and positron emission tomography (PET), is among the earliest cardiovascular imaging fields to incorporate machine learning techniques. Early research utilised artificial neural networks to analyse myocardial perfusion imaging and aid automated detection of coronary artery disease [20].

Recent advances in machine learning and deep learning have greatly enhanced the diagnostic and prognostic capabilities of nuclear cardiology. AI-driven analysis of myocardial perfusion imaging allows automated detection of coronary artery disease and improves the prediction of major adverse cardiovascular events and mortality [21]. Multicentre studies using deep learning applied to SPECT myocardial perfusion imaging have shown improved detection of obstructive coronary artery disease compared with traditional quantitative analysis methods [22].

Alongside diagnostic interpretation, AI has also been explored for optimising radiopharmaceutical imaging protocols and predicting tracer distribution, which may improve therapy planning and enable more personalised approaches in nuclear cardiovascular medicine [23].

Despite these advances, most AI applications in nuclear cardiology still rely on retrospective datasets and specialised imaging protocols. Broader multicentre validation and prospective evaluation are therefore needed before widespread clinical implementation. In addition, future studies should demonstrate whether improved diagnostic or prognostic performance translates into better clinical decision-making and patient outcomes.

#### 2.2.2. Echocardiography

Echocardiography is the most widely used imaging technique in cardiovascular medicine and has thus become a major focus of AI development. Early automation in echocardiography was limited to computer-assisted border detection and the measurement of ventricular volumes, including techniques such as speckle-tracking echocardiography, which rely on predefined correlation rules to track acoustic patterns between frames [24]. While these methods improved measurement reproducibility, they remained dependent on manual acquisition and operator expertise.

With the advancement of deep learning and the availability of large annotated imaging datasets, more sophisticated AI-based methods have emerged. Convolutional neural networks (CNNs) now enable automated view classification, chamber segmentation, and quantitative assessment of cardiac function with accuracy approaching that of expert interpretation [25,26]. In a large deep learning study involving over 200,000 echocardiographic images, CNNs achieved view classification accuracies of approximately 97–98% across standard echocardiographic views [26]. Similarly, deep learning–based segmentation models have demonstrated strong agreement with expert analysis, reaching a Dice similarity coefficient of about 0.92 for left ventricular segmentation [27].

AI-based echocardiographic analysis has also proven highly effective in the automated estimation of left ventricular ejection fraction. Video-based deep learning models have demonstrated mean absolute errors of approximately 4–5% in estimating ejection fraction compared to manual measurements [27]. These models can also identify regional wall motion abnormalities and classify heart failure phenotypes with high diagnostic accuracy [27,28].

Beyond automated measurements, AI-driven echocardiographic analysis can detect subtle myocardial abnormalities that may be difficult to recognise visually, aiding earlier identification of cardiomyopathies, cardiac amyloidosis, and cancer therapy–related cardiac dysfunction [29,30,31]. Additionally, deep learning approaches have demonstrated the potential to identify structural heart disease patterns directly from echocardiographic images and videos, emphasising AI’s ability to uncover imaging features beyond traditional interpretation [32].

Beyond measurement automation, AI is increasingly used to optimise the echocardiographic workflow. AI-assisted acquisition systems can guide probe positioning and support image quality optimisation during scanning. Automated analysis pipelines may reduce interobserver variability and shorten examination times, with some studies reporting workflow time reductions of up to 50% while maintaining diagnostic performance [33]. Furthermore, integrating echocardiographic imaging with clinical data, laboratory parameters, and other imaging modalities within multimodal AI frameworks has shown promise in improving diagnostic precision and prognostic risk stratification in conditions such as heart failure and valvular disease [34].

Although these are impressive technical achievements, most AI-based echocardiographic models have been developed and validated using retrospective or selected datasets, often from specialised centres with standardised acquisition protocols. Their performance in routine clinical practice may be affected by variable image quality, differences in ultrasound vendors, operator expertise, and patient characteristics. Consequently, broader external validation and prospective multicentre studies remain essential before widespread clinical adoption. Future research should also determine whether AI-assisted echocardiography improves diagnostic confidence, workflow efficiency, and patient outcomes beyond conventional expert interpretation. [25,26,27,33,34].

#### 2.2.3. Vascular Ultrasound

AI has also increasingly been utilised in vascular ultrasound imaging. Deep learning algorithms have been developed for automated vessel segmentation and carotid plaque characterisation, enabling reliable identification of plaque morphology and haemodynamically relevant stenoses with diagnostic performance comparable to expert interpretation [35,36]. In particular, CNNs have demonstrated high accuracy in classifying plaque composition, distinguishing among lipid-rich, fibrous, and calcified plaques, with reported classification accuracies of 85–90% on validation datasets [37,38].

Beyond plaque analysis, AI-based vascular ultrasound has demonstrated potential to identify arterial abnormalities, including aneurysmal dilatation and arterial narrowing. Automated screening systems for abdominal aortic aneurysm have achieved sensitivities above 90% in pilot studies, supporting the use of AI-assisted ultrasound for scalable vascular screening programmes [39]. AI-driven ultrasound analysis may also facilitate long-term monitoring of vascular disease progression by enabling automated quantification of plaque burden and vascular remodelling.

In venous imaging, machine learning models have also been explored for the automated detection of thrombotic patterns. These systems can assist in diagnosing conditions such as deep vein thrombosis by recognising characteristic flow and compressibility patterns on ultrasound imaging, thereby enhancing the efficiency of the diagnostic workflow and reducing examination duration [40].

More recently, advanced frameworks that combine reinforcement learning and explainable AI have been introduced to aid in ultrasound acquisition guidance and real-time image quality control. These systems can offer feedback during image acquisition and assist probe positioning, which may further decrease operator dependence and interobserver variability in vascular ultrasound examinations [41].

Even though these are promising developments, most AI applications in vascular ultrasound remain at the research stage and have been evaluated predominantly in small or highly selected cohorts. In addition, reliable plaque characterisation still depends on high-quality image acquisition and expert annotation, limiting generalisability across operators and clinical settings. Future studies should focus on prospective multicentre validation, integration into routine vascular screening programmes, and the development of explainable AI approaches to improve clinical interpretability and trust [36,37,39,42].

#### 2.2.4. Cardiac Magnetic Resonance

Cardiac magnetic resonance (CMR) is regarded as the gold standard for the quantitative assessment of cardiac structure and function, making it a key focus for artificial intelligence (AI) applications. Deep learning techniques have been employed throughout the CMR workflow, including image reconstruction, automated segmentation, and quantitative functional analysis [43,44].

AI-based reconstruction methods can significantly speed up image acquisition while maintaining diagnostic image quality, thereby reducing scan times and breath-hold requirements and enhancing patient comfort [43]. Recent deep learning reconstruction frameworks have shown acceleration factors of up to four- to eight-fold compared with traditional techniques, all while preserving diagnostic image fidelity [45].

Furthermore, deep learning algorithms enable automated segmentation of cardiac chambers with performance close to expert-level analysis. Several models have achieved Dice similarity coefficients above 0.90 for ventricular segmentation, enabling reliable and reproducible measurements of ventricular volumes and myocardial mass [46,47]. Similar deep learning methods have also been used with dynamic contrast-enhanced MRI, where automated segmentation of the ventricular myocardium across temporal frames allows for a more comprehensive functional assessment [48].

Beyond structural assessment, AI-driven analysis of myocardial tissue characteristics and deformation patterns has demonstrated the potential to detect pathological changes, including myocardial fibrosis, oedema, and perfusion abnormalities. These methods may enable improved phenotyping of cardiovascular diseases, including cardiomyopathies and inflammatory myocardial disorders, by extracting subtle imaging features not easily visible to human observers [44]. More recently, transformer-based architectures have been introduced for CMR analysis, enabling improved modelling of spatial and temporal relationships in cine and late gadolinium enhancement sequences. For example, a video-based Swin Transformer model has been validated for automated CMR screening and diagnosis across multiple cardiovascular diseases, illustrating the potential of transformer architectures beyond conventional CNN-based segmentation tasks [49].

Despite these advances, many AI applications in CMR have been developed using highly curated datasets from specialised centres with standardised imaging protocols. Broader external validation across different scanners, acquisition protocols, and patient populations remains necessary to ensure robust clinical performance. In addition, future studies should demonstrate whether AI-assisted CMR improves diagnostic efficiency, clinical decision-making, and patient outcomes beyond those achieved by expert interpretation.

#### 2.2.5. Cardiac Computed Tomography

Cardiac computed tomography (CT), especially coronary CT angiography (cCTA), has become a key area for the application of artificial intelligence (AI) in cardiovascular imaging. Deep learning–based algorithms enable automated coronary artery segmentation, plaque detection, and quantitative analysis of coronary atherosclerosis [50]. These methods enable rapid analysis of large imaging datasets and support comprehensive assessment of coronary plaque burden and morphology.

Moreover, AI-driven methods enable automated coronary artery calcium (CAC) scoring that closely aligns with traditional Agatston scoring, demonstrating high agreement with manual expert analysis and correlation coefficients above 0.95 in validation studies, thereby enhancing efficiency and consistency in cardiovascular risk assessment [51].

More advanced AI models can further characterise plaque composition and vascular remodelling, supporting improved risk stratification in patients with coronary artery disease. Deep learning techniques have demonstrated promising performance in identifying high-risk plaque features such as low-attenuation plaque and positive remodelling, which are associated with an increased risk of adverse cardiovascular events [52].

Machine learning models have also been developed for CT-derived fractional flow reserve (FFR-CT), enabling functional assessment of coronary lesions in addition to anatomical evaluation. AI-based FFR-CT approaches have demonstrated diagnostic performance comparable to computational fluid dynamics–based methods, with reported diagnostic accuracies around 80–85% for identifying haemodynamically significant stenoses [53,54].

Beyond coronary artery analysis, AI-based CT analysis has also been used for valvular heart disease. Deep learning algorithms have proven capable of automatically segmenting cardiac valves from CT angiography datasets, enabling automated evaluation of valvular morphology and supporting quantitative assessment of conditions such as aortic valve disease [55].

From a translational perspective, many AI-based CT applications have been evaluated primarily on retrospective datasets from specialised centres and may be affected by variations in scanner vendors, acquisition protocols, image quality, and patient populations. While automated calcium scoring and coronary plaque analysis are approaching routine clinical implementation, more advanced applications, including AI-guided plaque phenotyping and FFR-CT, require broader prospective multicentre validation and evidence of incremental clinical benefit over existing diagnostic workflows. Future studies should further assess the impact of AI-assisted CT on clinical decision-making, patient outcomes, and healthcare resource utilisation [56,57].

### 2.3. Artificial Intelligence in Paediatric Cardiovascular Imaging

Building on advances in AI for adult cardiovascular imaging, similar approaches have recently been adapted for paediatric cardiology. However, paediatric applications present additional challenges due to smaller cardiac structures, higher heart rates, congenital anatomical variability, and the limited availability of large annotated datasets [58]. These factors often require specialised model architectures and careful training strategies to ensure robust performance across heterogeneous paediatric populations.

Early developments have mainly focused on echocardiographic evaluation of congenital heart disease. Deep learning models have been developed for automated view classification, segmentation, and detection of structural abnormalities in foetal and paediatric echocardiography, achieving diagnostic performance comparable to expert interpretation, with reported classification accuracies of 85–90% in validation datasets [59,60,61].

Furthermore, AI-assisted three-dimensional reconstruction using CT and CMR datasets has been studied to support surgical planning in complex congenital heart conditions. These technologies enable enhanced visualisation of detailed anatomical relationships and assist preoperative assessment in conditions such as double-outlet right ventricle or transposition of the great arteries [60,62,63].

Beyond congenital malformations, AI has also been applied to paediatric vascular diseases. Deep learning algorithms have demonstrated promising results in detecting and measuring coronary artery aneurysms in Kawasaki disease, enabling automated assessment of coronary dimensions and enhanced monitoring of disease progression. Similarly, AI-assisted CMR segmentation can support the quantitative evaluation of aortic dimensions in paediatric aortopathies, potentially allowing earlier identification of progressive vascular dilatation in syndromic conditions such as Marfan syndrome [64].

Clinical translation in paediatric cardiovascular imaging remains particularly challenging because datasets are smaller, disease phenotypes are more heterogeneous, and congenital anatomy varies substantially between patients. Consequently, models trained at a single centre or on a single lesion group may not generalise reliably to other institutions, imaging protocols, or congenital heart disease subtypes. Future work should therefore prioritise multicentre paediatric datasets, transparent reporting of validation cohorts, and prospective evaluation of whether AI-assisted imaging improves diagnostic confidence, surgical planning, or longitudinal follow-up in children with cardiovascular disease [58,59].

### 2.4. Artificial Intelligence in Cardiovascular Surgery

Although AI applications in cardiovascular surgery are still less developed than those in diagnostic imaging, recent advancements show growing potential for intraoperative guidance and surgical planning. Improvements in image analysis and computational modelling have created new opportunities for surgical planning, intraoperative support, and perioperative risk assessment.

One of the earliest uses involves AI-assisted image segmentation and three-dimensional reconstruction, which enhance visualisation of complex anatomy and support preoperative planning in both congenital and adult cardiac surgery [63]. These technologies can enable the creation of patient-specific anatomical models and virtual surgical simulations, allowing surgeons to explore different procedural strategies before intervention. When combined with computational modelling, these approaches may also facilitate the simulation of haemodynamic changes and optimisation of surgical strategies prior to surgery [65].

During surgical procedures, AI-based computer vision systems are increasingly being investigated to identify anatomical landmarks and recognise surgical workflow steps in real time. Such systems may facilitate context-aware intraoperative assistance, automated documentation of surgical steps, and enhanced workflow analysis [66]. In vascular surgery, machine learning algorithms have also been employed in endovascular procedures, aiding with vessel segmentation, device sizing, and procedural planning for interventions such as endovascular aneurysm repair [67].

Beyond procedural guidance, AI-driven predictive models are increasingly utilised to estimate perioperative risk, including postoperative complications, reinterventions, and mortality. By integrating large clinical datasets with imaging and procedural variables, these models may enhance risk stratification and support personalised perioperative decision-making, sometimes outperforming traditional risk scores [68].

Following cardiovascular surgery, AI-supported clinical decision-support systems may facilitate remote post-discharge monitoring by integrating continuously collected physiological data with clinical information. Machine-learning analysis of wearable-derived biometric data has shown potential to identify postoperative complications before symptoms develop, while dedicated decision-support systems have been developed to support risk-based monitoring after cardiothoracic surgery. Such systems may enable earlier recognition of postoperative deterioration and more targeted follow-up. However, their clinical usefulness depends on adequate calibration, manageable false-positive rates, reliable data transmission, clearly defined escalation pathways, and evidence that alerts improve outcomes without increasing unnecessary clinical workload or readmissions [69,70].

These developments also align with the emerging concept of “twin transformation” in cardiothoracic surgery, in which digital innovation and environmental sustainability are considered together. AI-supported procedural planning, workflow optimisation, remote follow-up, and resource allocation may improve efficiency while potentially reducing avoidable travel, repeat investigations, material use, and hospital utilisation. However, the environmental effects of AI-enabled surgical pathways have rarely been evaluated directly, and future studies should assess clinical, organisational, and sustainability outcomes together [65].

Nevertheless, the clinical integration of AI into cardiovascular surgery remains in its early stages. Many currently available systems have been evaluated in proof-of-concept studies or at highly specialised centres, and their performance in complex intraoperative environments has yet to be established. In addition to technical accuracy, future research should address real-time robustness, surgeon–AI interaction, regulatory considerations, and the extent to which AI-assisted surgical guidance translates into improved procedural efficiency, patient safety, and clinical outcomes [71,72].

### 2.5. Multimodal Artificial Intelligence in Cardiovascular Medicine

A significant recent advancement in artificial intelligence (AI) for cardiovascular medicine is the development of multimodal AI systems that integrate information from various heterogeneous data sources. These systems combine inputs such as imaging data, physiological signals, laboratory values, electronic health records, and genomic information. Integration can be achieved through different computational strategies, including early feature fusion, where features from diverse modalities are combined before model training, or late decision fusion, where predictions from individual modalities are subsequently aggregated [73,74,75,76].

While early AI applications in cardiology were largely limited to single data modalities such as electrocardiography or medical imaging, contemporary models increasingly integrate imaging data, physiological signals, clinical records, laboratory results, and genomic information within unified predictive frameworks [73,77]. By combining complementary information across modalities, multimodal AI systems can capture complex interactions between anatomical, functional, and molecular aspects of cardiovascular disease.

Recent studies show that multimodal approaches can enhance diagnostic accuracy and improve the prediction of clinically relevant outcomes, including disease progression and major adverse cardiovascular events (MACE). Models that combine imaging-derived features with clinical variables and laboratory biomarkers have demonstrated better prognostic performance compared to single-modality approaches [78,79].

However, multimodal models should be evaluated against a clearly defined clinical use case and appropriate simpler clinical or unimodal comparators. The incremental contribution of each modality should be quantified, as apparent performance gains may reflect unequal data availability, informative missingness, or data leakage rather than genuine complementary value.

Beyond clinical prediction, multimodal AI also increasingly supports clinical decision-making. AI systems that combine imaging findings with electronic health records and patient-specific risk factors can assist physicians in treatment planning and therapeutic choices [80,81]. Such approaches mark an important step towards clinically integrated AI systems that support physicians throughout various stages of cardiovascular care.

Another significant development is the integration of genomic and molecular data with imaging and physiological signals. These methods enable the exploration of genotype–phenotype relationships in cardiovascular disease and may support improved risk stratification and more personalised treatment strategies by connecting molecular pathways with structural and functional cardiac phenotypes [76,82].

The rapid growth of digital health technologies further enhances the potential of multimodal AI systems. Wearable sensors, remote monitoring platforms, and interoperable electronic health record systems produce large-scale longitudinal datasets that can be integrated into AI models for ongoing cardiovascular risk assessment and disease monitoring [79]. These advancements enable AI systems to shift from episodic diagnostics to dynamic monitoring of cardiovascular health trajectories.

Emerging multimodal architectures are also moving beyond conventional feature-fusion approaches. Transformer-based models can process sequential and high-dimensional data, such as ECG waveforms, imaging series, and longitudinal electronic health record trajectories, while graph neural networks can model relational structures, including patient similarity networks, molecular interaction networks, and genotype–phenotype relationships. In parallel, large language models and generative AI systems may offer new interfaces for clinical documentation, literature synthesis, decision support, and patient communication. However, these approaches remain in the early stages of cardiovascular medicine and require careful evaluation because of risks such as hallucinations, bias, limited explainability, and uncertain regulatory status [74,82,83].

From a digital health perspective, the success of multimodal AI relies heavily on interoperability among diverse data infrastructures. Standardised data exchange frameworks, scalable health data platforms, and strong governance structures are therefore vital prerequisites for implementing multimodal AI in routine clinical practice [84]. In addition, missing data, inconsistent coding practices, heterogeneous imaging protocols, and differences between electronic health record systems can degrade model performance and limit transferability across institutions. Transparent reporting of modality availability, missing-data handling, temporal separation of training and validation data, and modality-specific ablation analyses is therefore important for assessing transportability and genuine incremental value. Future multimodal AI systems will therefore require external validation, transparent reporting, data harmonisation strategies, and continuous post-deployment monitoring to ensure reliable and equitable clinical performance.

Although the preceding sections focus primarily on clinical domains, these applications rely on distinct methodological foundations. Early cardiovascular AI systems were largely based on rule-based or traditional machine-learning approaches, whereas contemporary applications increasingly use deep learning architectures for image, signal, and multimodal data analysis. More recent developments include transformer-based models, graph neural networks, generative AI, reinforcement learning, and privacy-preserving approaches such as federated learning. The major AI methodologies underpinning these cardiovascular applications are summarised in Table 1, spanning from early rule-based systems to contemporary multimodal, generative, and privacy-preserving approaches.

Traditional machine-learning models remain well suited to structured clinical data and smaller datasets and may offer greater interpretability, but they depend on manual feature engineering. Convolutional neural networks have achieved the greatest maturity in image and signal analysis, although their performance may decline across devices, acquisition protocols, and institutions. Transformer-based and graph-based approaches can model longer-range, temporal, and relational information, but generally require larger datasets and greater computational resources and remain less extensively validated in cardiovascular practice. Large language and generative models provide flexible interfaces for documentation, education, and decision support, but hallucination, bias, limited explainability, and uncertain clinical accountability currently restrict their use. Multimodal models may offer incremental performance by integrating complementary data sources, although this benefit is not consistently established over simpler clinical or unimodal models and may be affected by missingness, leakage, and limited transportability.

### 2.6. System-Level Applications of Artificial Intelligence in Cardiovascular Medicine

Beyond imaging and interventional uses, AI is increasingly shaping the wider field of cardiovascular medicine, including medical education, clinical decision-making, workflow management, and clinical research. As digital cardiovascular health ecosystems grow, AI systems more often integrate with electronic health records, wearable devices, telemedicine platforms, and large-scale clinical databases, enabling more cohesive and data-driven healthcare delivery [2,84].

In medical education and training, AI-powered simulation and adaptive learning platforms enable physicians and surgeons to develop procedural, interpretive, and cognitive skills within realistic, feedback-rich environments. Virtual and augmented reality systems equipped with AI assessment algorithms can objectively evaluate technical performance and offer personalised guidance, thus reducing learning curves and enhancing long-term skill retention [85,86,87]. Computer vision–based systems have also been investigated for analysing surgical workflows and operator performance in cardiothoracic procedures, allowing for objective assessment of technical proficiency and procedural efficiency [66]. In cardiovascular imaging training, AI-supported tools have demonstrated improvements in echocardiographic interpretation accuracy and facilitate scenario-based learning environments for clinicians [87]. Additionally, emerging generative AI models such as large language systems may support trainees in knowledge retrieval, case-based learning, and exam preparation, creating a new interface between AI and medical education [83,85].

In clinical practice, AI-powered systems are increasingly serving as decision-support and workflow-optimisation tools. By combining multimodal patient data, including imaging, physiological signals, laboratory results, and clinical records, these systems can aid in real-time diagnosis, risk prediction, and therapeutic decision-making [80,81]. AI-supported triage algorithms may improve patient throughput and decrease diagnostic delays in acute cardiovascular care, while predictive models can assist with patient prioritisation, imaging workflow management, and procedural scheduling [80,88]. Furthermore, AI-enabled predictive analytics may facilitate early identification of high-risk patients and enable more proactive strategies for disease management within large healthcare systems [2,89,90].

A rapidly evolving area is the use of AI in cardiovascular clinical trials, where it has the potential to transform trial design, recruitment, and analysis. Machine learning algorithms can enable automated patient identification from large clinical, imaging, and genomic databases, allowing for more efficient and representative cohort selection [91,92]. AI-enabled monitoring platforms can continuously analyse physiological signals and imaging biomarkers obtained from wearable devices or remote monitoring systems, thus supporting adaptive trial designs that modify sample size, endpoints, or therapeutic strategies based on interim results [91,92,93]. Additionally, natural language processing techniques may facilitate automated endpoint adjudication and data harmonisation across multicentre trials, reducing administrative workload while enhancing consistency and transparency in clinical research [91,92,94].

However, system-level AI applications face particularly high implementation barriers because they must operate within complex clinical workflows rather than in isolated diagnostic tasks. Evidence of improved predictive performance alone is therefore insufficient. Future studies should demonstrate measurable benefits for patient outcomes, clinician workload, diagnostic efficiency, resource utilisation, and healthcare costs. Broader adoption will additionally depend on reimbursement pathways, clinician acceptance, local implementation resources, and evidence that workflow gains justify acquisition, integration, and maintenance costs. In addition, successful implementation will require prospective evaluation, user-centred design, post-deployment monitoring, and clear accountability structures to ensure that AI systems support rather than disrupt clinical decision-making [77,95,96].

The major domains and representative clinical applications of artificial intelligence in cardiovascular medicine discussed throughout this review are summarised in Table 2.

## 3. Clinical Maturity, Future Directions and Challenges

To complement the application-oriented overview in Table 2, Table 3 and Table 4 distinguish between the validation status and the translational implementation of major AI applications in cardiovascular medicine. Table 3 summarises technical validation, external validation, prospective evidence, and the principal remaining evidence gaps. Table 4 separately addresses regulatory or commercial availability, actual clinical implementation, demonstrated workflow or clinical benefit, and the main barriers to broader translation. This distinction is important because strong technical performance, external validation, regulatory authorisation, commercial availability, routine clinical use, and demonstrated patient benefit represent different stages of translation and should not be treated as equivalent.

Across cardiovascular AI, technical development has generally progressed more rapidly than clinical translation. High performance on retrospective or benchmark datasets does not necessarily imply successful external validation, prospective utility, regulatory authorisation, routine implementation, or improved patient outcomes. These stages therefore represent distinct dimensions of maturity and should not be treated as equivalent. The following discussion addresses these gaps across the major domains reviewed.

The corresponding translational and implementation status of these applications is summarised separately in Table 4.

Across cardiovascular AI, retrospective measures of discrimination, classification accuracy, or segmentation performance remain more commonly reported than prospective evidence of clinical utility. Calibration, false-positive consequences, workflow effects, patient outcomes, and cost-effectiveness are often incompletely assessed. Accordingly, strong technical performance should be viewed as only one component of translation, not as evidence of clinical benefit in itself.

Despite rapid advances in artificial intelligence (AI) in cardiovascular medicine, several challenges remain before these technologies can be seamlessly integrated into routine clinical practice. As summarised in Table 3 and Table 4, clinical maturity varies substantially across domains: while selected applications, such as AI-assisted arrhythmia detection, echocardiographic ejection fraction estimation, cardiac MRI segmentation, and CT-based calcium scoring, are approaching routine clinical use, many multimodal, paediatric, surgical, and system-level applications remain in the emerging or investigational stages. A central limitation across domains is reliance on highly curated retrospective datasets from specific institutions or research cohorts. Consequently, model performance may deteriorate when algorithms are applied to more diverse patient populations, imaging protocols, device platforms, disease prevalences, or healthcare systems. Robust external validation and generalisability across geographic regions, clinical settings, and patient demographics are therefore essential prerequisites for dependable clinical deployment [77,95,96,97].

Algorithm transparency and interpretability pose additional challenges to widespread clinical adoption. Many deep learning models function as so-called “black boxes,” hindering clinicians’ understanding of the underlying reasoning behind specific predictions or classifications. Enhancing explainability through interpretable model architectures, saliency mapping techniques, and transparent reporting standards may help boost clinician trust and support regulatory approval and clinical integration [95,96]. As AI systems become more adaptive and are deployed across evolving clinical environments, post-deployment monitoring will become increasingly important. Model drift, shifts in patient populations, evolving clinical practice, and variations in device hardware or software may all degrade model performance over time. Continuous performance assessment and safe mechanisms for model updating will therefore be essential to maintaining reliability after clinical deployment.

The responsible deployment of AI systems also requires careful consideration of data governance, privacy, and regulatory oversight. Large-scale datasets derived from imaging platforms, electronic health records, and wearable devices form the foundation of many AI applications, yet their use raises important concerns regarding patient privacy, data security, and ethical data sharing. Regulatory agencies and professional societies are therefore increasingly developing frameworks for the evaluation, validation, and monitoring of AI-based medical technologies [77,96,98]. In this context, transparent reporting standards, auditability, cybersecurity safeguards, and clearly defined accountability structures will be necessary to ensure the safe integration of AI tools into cardiovascular workflows.

An additional concern is the potential for algorithmic bias. AI models trained on limited or unrepresentative datasets may perform less accurately in underrepresented patient populations, potentially exacerbating existing healthcare disparities. Recent studies have also demonstrated that large language models used in medical communication may reproduce or amplify biases present in training data, raising concerns about fairness, transparency, and the responsible use of generative AI in healthcare settings [99]. Ensuring equitable model performance across diverse patient populations and healthcare systems will therefore be essential for the responsible integration of AI into cardiovascular medicine [96,100]. This will require not only more diverse training and validation datasets, but also subgroup performance reporting, fairness evaluation, and ongoing surveillance after implementation.

Ultimately, the successful integration of AI in cardiovascular care will depend on close interdisciplinary collaboration among clinicians, engineers, data scientists, and regulatory authorities. Rather than replacing clinical expertise, AI is more likely to serve as a complementary tool that enhances physician decision-making and enables more precise, data-driven cardiovascular treatment [96]. The most clinically valuable systems will therefore be those that combine robust technical performance with interpretability, workflow compatibility, prospective validation, and demonstrable benefit for patients and healthcare systems.

## 4. Limitations

This review has several limitations that should be acknowledged. As a narrative review based on a selective synthesis of relevant literature identified primarily through PubMed and citation tracking, it does not claim to be systematically comprehensive. Studies were selected for clinical relevance, methodological influence, and representativeness rather than through exhaustive database screening, which may introduce selection bias towards higher-visibility publications.

Second, the breadth of this review, spanning electrocardiography, multiple imaging modalities, paediatric and surgical applications, multimodal AI, and system-level applications, necessarily limits the depth of discussion within each domain. More focused reviews within each subdomain will provide greater granularity than a single integrative synthesis.

Third, the field of cardiovascular AI is evolving rapidly. Given publication timelines, some developments emerging after the literature search may not be reflected in this review.

Finally, this is a single-author narrative review and therefore reflects a single integrative perspective. While this may limit the breadth of disciplinary viewpoints, it also allows a coherent conceptual synthesis across the historical and translational development of cardiovascular AI.

## 5. Conclusions

Artificial intelligence has advanced rapidly from early rule-based electrocardiogram interpretation systems to sophisticated multimodal models that integrate diverse cardiovascular data sources. Across electrocardiography, cardiovascular imaging, surgical planning, and healthcare system applications, AI increasingly supports earlier disease detection, refined risk stratification, clinical decision-making, and workflow optimisation.

The next phase of development will likely be shaped by the integration of AI into broader digital health ecosystems, including wearable monitoring devices, remote patient management systems, and interoperable health data infrastructures. These developments enable continuous data collection and real-time analysis, opening new possibilities for proactive cardiovascular monitoring and prevention.

Despite these advances, significant challenges remain. Reliable prospective validation, algorithmic transparency, data governance, and regulatory oversight will be crucial to ensuring the safe and fair deployment of AI-based technologies in clinical practice.

If successfully integrated into clinical workflows, AI has the potential to reshape cardiovascular medicine by enabling more precise diagnostics, improved risk stratification, and data-driven healthcare delivery. Ultimately, the convergence of artificial intelligence and digital cardiovascular health may foster a shift from episodic, modality-based assessment to more continuous, preventive, risk-adapted, and personalised models of cardiovascular care.

## Figures and Tables

**Table 1:** Overview of major artificial intelligence methodologies and their applications in cardiovascular medicine.

AI Method	Architecture/Examples	Typical CV Application	Key Strengths	Key Limitations
Rule-Based Systems	Expert systems, decision trees	Early ECG interpretation, CAD detection thresholds	Transparent, interpretable, no training data needed	Rigid rules; poor adaptability to complex patterns
Traditional Machine Learning	SVM, Random Forest, Gradient Boosting	Risk stratification, structured clinical data	Effective on tabular data; interpretable features	Requires manual feature engineering; limited to raw images
Convolutional Neural Networks (CNN)	ResNet, U-Net, EfficientNet	Image segmentation, view classification, EF estimation	High accuracy on image tasks; scalable	Requires large annotated datasets; black-box decision-making
Recurrent Neural Networks/LSTM	RNN, LSTM, GRU	ECG time-series, longitudinal EHR modelling	Captures temporal dependencies in sequential data	Vanishing gradient; computationally intensive
Transformer-Based Models	Vision Transformer (ViT), Swin Transformer	CMR analysis, multimodal data integration	Long-range context modelling; strong multimodal fusion	High computational cost; requires large datasets
Graph Neural Networks (GNNs)	GCN, GAT	Patient similarity networks, genotype–phenotype mapping	Models relational and network structures	Limited cardiovascular validation; complex implementation
Large Language Models (LLM)	GPT-4, Med-PaLM, clinical LLMs	Clinical documentation, decision support, education	Flexible natural language interface; knowledge synthesis	Hallucination risk; bias; regulatory uncertainty; limited cardiac-specific validation
Generative AI	GANs, Diffusion Models, VAEs	Synthetic data augmentation, image reconstruction	Addresses data scarcity; accelerates MRI acquisition	Output quality variable; potential for misleading synthetic data
Multimodal Fusion Models	Late/early fusion, cross-modal transformers	Imaging + EHR + genomics + ECG integration	Captures complementary information across modalities	Data harmonisation challenges; interoperability barriers; limited prospective validation
Reinforcement Learning	Deep Q-Networks, policy gradient methods	Ultrasound acquisition guidance, adaptive protocols	Learns optimal sequential decision strategies	Sample inefficiency; safety concerns in clinical deployment
Federated/Privacy-Preserving AI	Federated Learning, Swarm Learning	Multicentre model training without data sharing	Preserves privacy; enables large-scale collaboration	Data heterogeneity; convergence challenges; governance complexity

**Table 2:** Major domains and clinical applications of artificial intelligence in cardiovascular medicine.

Domain	Subdomain/Method	Typical AI Tasks	Clinical Applications
**Electrocardiography and -physiology**	Electrocardiography	Signal classification, pattern recognition [5,6,8]	Arrhythmia detection, AF screening, LV dysfunction prediction
	Cardiac Electrophysiology	Signal classification, substrate characterisation, target localisation, outcome prediction [14,15,16,17]	Mapping support, ablation planning, recurrence prediction
**Cardiovascular Imaging**	Echocardiography	View classification, segmentation, functional quantification [26,27,33]	Ejection fraction estimation, cardiomyopathy detection
	Nuclear imaging (SPECT/PET)	Perfusion analysis, outcome prediction [21,22]	CAD detection, risk stratification
	Vascular ultrasound	Plaque segmentation, plaque composition analysis [37,39]	Stroke risk assessment, carotid disease monitoring
	Cardiac MRI (CMR)	Chamber segmentation, tissue characterisation [47,49]	Ventricular volume analysis, fibrosis detection
	Cardiac CT	Plaque detection, CAC scoring, FFR-CT [51,53]	CAD and risk stratification
**Paediatric cardiovascular imaging**	Congenital heart disease imaging	Automated view classification, anatomical reconstruction [58,59]	Surgical planning, congenital anomaly detection
**Cardiovascular surgery**	Surgical planning and intraoperative support	Image segmentation, computer vision [66,68]	Preoperative planning, intraoperative guidance
**Multimodal AI**	Multimodal data fusion	Integration of imaging, EHR, physiological signals, genomics [73,74,82]	Risk prediction, treatment response prediction
**System-level applications**	Clinical decision support	Predictive modelling, risk stratification [80,81]	Diagnostic support, treatment planning
	Medical education	Simulation systems, AI-guided training [85,87]	Skills training, performance assessment
	Clinical trials	Patient identification, endpoint analysis [91,92]	Recruitment optimisation, adaptive trial design

**Table 3:** Validation and evidence status of major AI applications in cardiovascular medicine.

Subdomain	Application	Technical Validation	External Validation	Prospective Evidence	Main Evidence Gap
**Electro-Cardiography**	Arrhythmia detection/AF screening [4,5,6,7,8,10,11]	Strong diagnostic performance has been demonstrated in large annotated ECG datasets.	External and population-based validation is available for selected applications.	Prospective and real-world evidence remains limited and varies by intended use.	Effects on downstream testing, false-positive burden, therapeutic decisions, and patient outcomes remain incompletely established.
	LV dysfunction prediction [8,9]	Promising retrospective discrimination has been demonstrated in selected populations.	Some external validation is available, but performance may vary across acquisition systems and populations.	Prospective pathway-based evaluation is limited.	Incremental value over conventional screening and the effect on treatment or outcomes remain uncertain.
	Electrolyte/extracardiac detection [12,13]	Proof-of-concept performance has been demonstrated for selected physiological and extracardiac conditions.	Independent external validation is limited.	Prospective clinical evidence is minimal.	Intended populations, disease prevalence, false-positive consequences, and clinical utility remain unresolved.
**Electrophysiology**	Intracardiac electrogram analysis, electroanatomical mapping, ablation support, and outcome prediction [14,15,16,17]	Promising technical performance has been demonstrated for arrhythmia classification, substrate characterisation, target localisation, and recurrence prediction.	External validation across centres, mapping systems, and signal-acquisition platforms remains limited	Prospective procedural and outcome evidence is scarce.	Platform dependence, heterogeneous signal acquisition, workflow integration, and incremental value over established mapping and ablation strategies remain insufficiently established.
**Nuclear (SPECT/PET)**	Perfusion analysis/CAD detection [20,21,22,23]	Machine-learning and deep-learning models have demonstrated strong retrospective diagnostic performance.	Multicentre validation is available for selected SPECT applications.	Prospective clinical evaluation remains limited.	Incremental benefit over established quantitative software and expert interpretation requires further evaluation.
	Prognostic/MACE prediction [21,22,23]	Registry-based and meta-analytic evidence supports improved risk discrimination.	External validation is heterogeneous across protocols and populations.	Prospective outcome evidence is limited.	Calibration, transportability, and effects on clinical management or outcomes remain insufficiently established.
**Echocardiography**	View classification and EF estimation [25,26,27,32,33]	Strong performance and reproducibility have been demonstrated for automated view classification, segmentation, and EF estimation.	Multicentre and independent validation is available for selected systems.	Prospective workflow and clinical evaluation are emerging but remain limited relative to retrospective evidence.	Evidence of improved patient outcomes, downstream decision-making, and cost-effectiveness remains limited.
	Cardiomyopathy, amyloidosis, and structural disease detection [28,29,30,31,32,34]	Promising diagnostic performance has been demonstrated in retrospective and selected multicentre cohorts.	Some external validation is available.	Prospective disease-specific evaluation remains limited.	Generalisability across vendors, referral settings, disease prevalence, and patient populations remains uncertain.
	Workflow optimisation and automated quantification [25,26,27,32,33,34]	Automated acquisition support and quantitative analysis are technically feasible and reproducible.	External validation is available for selected tools.	Prospective workflow studies remain limited.	Effects on clinician workload, examination quality, downstream testing, resource utilisation, and costs require further study.
**Vascular Ultrasound**	Carotid plaque characterisation [35,36,37,38]	Promising segmentation and plaque-classification performance has been demonstrated.	External validation remains limited.	Prospective evidence is scarce.	Dependence on acquisition quality, operator technique, annotation standards, and device characteristics limits generalisability.
	Abdominal aortic aneurysm screening [39,42]	High sensitivity has been reported in pilot evaluations.	Independent multicentre validation is limited.	Early prospective evidence is available.	Population-level screening performance, false-positive consequences, and effects on clinical pathways remain insufficiently characterised.
**Cardiac MRI (CMR)**	Chamber segmentation/EF [43,46,47,48,49]	Strong benchmark performance, frequently approaching expert reproducibility, has been demonstrated.	External and multicentre validation is available for selected models.	Prospective implementation evidence remains limited.	Failure modes in complex anatomy and evidence of improved clinical decisions or patient outcomes remain incompletely characterised.
	Tissue characterisation (LGE/T1/T2) [44,48,49]	Promising performance has been demonstrated for myocardial tissue analysis and disease classification.	External validation remains heterogeneous across scanners, protocols, and disease groups.	Prospective disease-specific evidence is limited.	Standardisation, calibration, and incremental value over expert interpretation remain insufficiently established.
	Accelerated reconstruction [45]	Technical feasibility and substantial acceleration have been demonstrated while preserving image quality.	Multi-vendor and cross-protocol validation remains limited.	Evidence is derived mainly from technical and feasibility studies.	Robustness, artefact detection, failure-mode characterisation, and clinical outcome impact require further study.
**Cardiac CT**	CAC scoring [51,56,57]	Strong agreement with manual scoring has been demonstrated.	Multicentre validation across different CT protocols is available.	Prospective implementation evidence remains limited.	Incremental effects on preventive treatment decisions, downstream testing, outcomes, and costs remain incompletely established.
	Coronary plaque quantification [50,52,56,57]	Strong technical performance and quantitative reproducibility have been reported.	Multicentre validation is available for selected systems.	Prospective evaluation remains limited.	Harmonisation across scanners and software platforms and demonstration of incremental clinical benefit are still required.
	FFR-CT/ischemia prediction [53,54,56,57]	Good diagnostic performance has been demonstrated against computational or invasive reference standards.	Multicentre validation is available.	Prospective multicentre evidence is more advanced than for many other cardiovascular AI applications, although still heterogeneous.	Effects on outcomes, workflow, resource utilisation, and cost-effectiveness require further clarification.
	Valvular assessment (TAVI planning) [55,56,57]	Automated valve segmentation and anatomical assessment are technically feasible.	External and multi-vendor validation remains limited.	Early prospective evidence is limited.	Robustness across complex anatomy, valve platforms, and calcification patterns remains uncertain.
**Paediatric Imaging**	CHD view classification/segmentation [58,59,60,61,62,63]	Promising technical performance has been demonstrated in selected congenital lesion groups.	External validation is limited by small and heterogeneous datasets.	Prospective evidence is minimal.	Dataset scarcity, anatomical heterogeneity, lesion-specific training, and cross-centre transportability remain major barriers.
	Kawasaki/aortopathy monitoring [61,64]	Proof-of-concept performance has been demonstrated for selected vascular abnormalities.	Independent validation is limited or absent.	Prospective longitudinal evidence is minimal.	Clinical thresholds, reproducibility over time, and effects on management remain uncertain.
**Cardiovascular Surgery**	Preoperative planning/3D reconstruction [63,65,66,71,72]	Technical feasibility has been demonstrated in specialised centres and selected complex cases.	External comparative validation is limited.	Evidence is based mainly on feasibility and observational studies.	Comparative evidence for improved planning, procedural safety, efficiency, or outcomes remains limited.
	Perioperative risk prediction [65,68,71,72]	Retrospective models have demonstrated promising predictive performance.	Some external or multicentre validation is available for selected applications.	Prospective evaluation is limited.	Calibration, workflow integration, transportability, and incremental value over established risk scores remain uncertain.
**Multimodal AI**	Imaging + EHR + genomics integration [73,74,75,76,77,78,79,82]	Retrospective studies indicate potential gains in diagnostic or prognostic performance.	Independent external validation remains limited.	Prospective evidence is rare.	Missing data, modality contribution, data leakage, interoperability, and incremental value over simpler models remain insufficiently addressed.
	Transformer/GNN/LLM approaches [49,74,77,82,83]	Strong technical promise has been demonstrated in selected tasks.	Cardiovascular-specific external validation is limited.	Prospective clinical evidence is minimal.	Computational burden, explainability, hallucination, bias, reproducibility, and transportability remain unresolved.
**System-Level AI**	Clinical decision support/triage [77,80,81,88,89,90]	Promising retrospective or simulated performance has been reported.	Independent external validation is limited and application-specific.	Prospective and pragmatic evidence remains limited.	Effects on clinician behaviour, diagnostic error, workflow, patient safety, and clinical outcomes remain uncertain.
	Medical education/simulation [85,87]	Feasibility and improvements in selected educational outcomes have been demonstrated.	Validation across institutions and learner groups is limited.	Some prospective educational evaluations are available.	Durability of learning effects and transfer to real-world clinical performance remain uncertain.
	Clinical trial support [91,92]	Technical feasibility has been demonstrated for NLP, recruitment, monitoring, and data harmonisation.	Independent validation across trial settings remains limited.	Prospective implementation evidence is limited.	Regulatory acceptability, standardisation, auditability, representativeness, and impact on trial quality remain insufficiently established.

Technical validation refers to performance demonstrated on development, benchmark, or task-specific datasets. External validation refers to evaluation in independent populations, institutions, devices, vendors, or healthcare settings. Prospective evidence includes prospective observational, pragmatic, or randomised clinical evaluations. These dimensions are presented separately because technical performance does not necessarily imply transportability, clinical implementation, or demonstrated patient benefit.

**Table 4:** Translational and implementation status of major AI applications in cardiovascular medicine.

Subdomain	Application	Regulatory or Commercial Status	Clinical Implementation	Demonstrated Clinical or Workflow Benefit	Main Translational Gap
**Electro-Cardiography**	Arrhythmia detection/AF screening [4,5,6,7,8,10,11]	Regulatory-cleared wearable and mobile ECG systems are available for selected rhythm-monitoring indications.	Used in selected screening, ambulatory monitoring, and remote-surveillance pathways.	Improved scalability of rhythm detection and prolonged monitoring has been demonstrated.	Effects on downstream testing, treatment decisions, false-positive burden, and patient outcomes remain variable across use cases.
	LV dysfunction prediction [8,9]	Selected investigational and commercial tools have been developed, but broad regulatory implementation remains limited.	Mainly used in research or selected specialist settings.	Potential for earlier identification of otherwise unrecognised ventricular dysfunction has been demonstrated.	Clinical pathways, confirmatory testing strategies, and effects on treatment or outcomes remain insufficiently established.
	Electrolyte/extracardiac detection [12,13]	No broadly established regulatory or commercial pathway.	Research use.	Clinical benefit has not been demonstrated.	Intended populations, false-positive consequences, downstream testing, and incremental value over conventional assessment remain unresolved.
**Electrophysiology**	Intracardiac electrogram analysis, electroanatomical mapping, ablation support, and outcome prediction [14,15,16,17]	Selected commercial and investigational decision-support tools are emerging; regulatory status remains application-specific.	Mainly research, pilot, or specialist-centre use.	Potential support for substrate identification, procedural planning, and recurrence prediction has been reported.	Prospective comparative evidence for improved procedural efficiency, safety, freedom from recurrent arrhythmia, and patient outcomes remains limited.
**Nuclear (SPECT/PET)**	Perfusion analysis/CAD detection [20,21,22,23]	Vendor-assisted and commercial quantitative analysis tools are available for selected applications.	Used in selected nuclear cardiology workflows, generally as decision support rather than autonomous interpretation.	Improved diagnostic consistency and automated image analysis have been reported.	Incremental benefit over established quantitative software and expert interpretation, together with effects on outcomes and resource use, remains uncertain.
	Prognostic/MACE prediction [21,22,23]	Limited commercial implementation.	Predominantly research and registry use.	Improved prognostic discrimination has been reported in retrospective datasets.	Evidence that model predictions alter management or improve outcomes remains limited.
**Echocardiography**	View classification and EF estimation [25,26,27,32,33]	Commercial and selected regulatory-cleared tools are available for automated acquisition, segmentation, and quantification.	Increasingly incorporated into selected echocardiographic workflows.	Improved reproducibility, automated quantification, and reduced analysis time have been demonstrated.	Patient-level outcome benefit, cost-effectiveness, and performance in suboptimal or heterogeneous imaging conditions require further evaluation.
	Cardiomyopathy, amyloidosis, and structural disease detection [28,29,30,31,32,34]	Selected commercial and investigational tools are available, but implementation remains indication-specific.	Mainly used in specialist or research settings.	Potential improvement in case detection and phenotyping has been demonstrated.	Effects on referral pathways, confirmatory testing, treatment decisions, and outcomes remain insufficiently established.
	Workflow optimisation and automated quantification [25,26,27,32,33,34]	Commercial acquisition-guidance and quantification systems are available.	Increasing but heterogeneous adoption across centres and vendors.	Reduced interobserver variability and shorter acquisition or analysis times have been reported.	Net effects on clinician workload, examination quality, resource utilisation, and cost remain incompletely characterised.
**Vascular Ultrasound**	Carotid plaque characterisation [35,36,37,38]	Limited commercial availability; most systems remain research-oriented.	Mainly used in research and specialist centres.	Automated plaque segmentation and more reproducible quantitative assessment have been demonstrated.	Clinical thresholds, cross-device robustness, effects on treatment decisions, and incremental value over expert interpretation remain uncertain.
	Abdominal aortic aneurysm screening [39,42]	Pilot and investigational systems are available.	Early or pilot implementation.	Potential improvement in screening efficiency and automated case detection has been demonstrated.	Population-level performance, false-positive consequences, training requirements, and cost-effectiveness remain unclear.
**Cardiac MRI (CMR)**	Chamber segmentation/EF [43,46,47,48,49]	Vendor-integrated and commercial automated analysis tools are available.	Increasingly used in selected routine CMR workflows.	Improved reproducibility and reduced manual segmentation time have been demonstrated.	Failure detection, complex anatomy, downstream decision-making, and patient-outcome effects require further evaluation.
	Tissue characterisation (LGE/T1/T2) [44,48,49]	Selected commercial and research tools are available.	Limited specialist-centre implementation.	More automated and reproducible tissue quantification and disease classification have been reported.	Standardisation across scanners and protocols and evidence of incremental clinical benefit remain limited.
	Accelerated reconstruction [44,45]	Vendor-integrated reconstruction systems are emerging.	Early clinical adoption in selected centres.	Shorter acquisition and reconstruction times with preserved image quality have been demonstrated.	Robustness, artefact recognition, failure-mode transparency, and effects on diagnostic decisions remain incompletely established.
**Cardiac CT**	CAC scoring [51,56,57]	Commercial and selected regulatory-cleared tools are available.	Used in selected cardiac and opportunistic CT workflows.	Improved efficiency, reproducibility, and automated identification of coronary calcification have been demonstrated.	Effects on preventive treatment initiation, adherence, downstream testing, patient outcomes, and cost-effectiveness remain incompletely established.
	Coronary plaque quantification [50,52,56,57]	Commercial plaque-analysis platforms are available.	Increasing use in selected specialist centres.	More rapid and reproducible plaque quantification and phenotyping have been demonstrated.	Harmonisation across platforms and evidence that quantitative plaque analysis improves management or outcomes remain limited.
	FFR-CT/ischemia prediction [53,54,56,57]	Commercial and regulatory-cleared platforms are available in selected healthcare systems.	Incorporated into selected diagnostic pathways for coronary artery disease.	Improved functional assessment from non-invasive CT and potential reduction in unnecessary invasive testing have been reported.	Availability, workflow integration, reimbursement, cost-effectiveness, and effects on long-term outcomes remain heterogeneous.
	Valvular assessment (TAVI planning) [55,56,57]	Commercial and vendor-integrated planning tools are emerging.	Used selectively in structural-heart and procedural-planning programmes.	Faster and more reproducible anatomical measurements have been reported.	Performance in complex anatomy and evidence of improved procedural safety or patient outcomes remain limited.
**Paediatric Imaging**	CHD view classification/segmentation [58,59,60,61,62,63]	No broadly established commercial or regulatory pathway.	Research and selected specialist-centre use.	Potential improvement in anatomical visualisation, segmentation, and surgical planning has been demonstrated.	Small datasets, lesion heterogeneity, workflow integration, and evidence of clinical benefit remain major limitations.
	Kawasaki/aortopathy monitoring [61,64]	No established commercial pathway.	Research use.	Potential for automated vascular measurement and lesion detection has been demonstrated.	Longitudinal reliability, clinical thresholds, management impact, and outcome benefit remain uncertain.
**Cardiovascular Surgery**	Preoperative planning/3D reconstruction [63,65,66,71,72]	Specialised commercial and institution-developed systems are available.	Selective use in complex congenital and adult surgical cases.	Improved anatomical visualisation and preoperative understanding have been reported.	Comparative evidence for reduced operative time, improved safety, better procedural decisions, or improved outcomes remains limited.
	Perioperative risk prediction [65,68,71,72]	Selected institutional and commercial predictive systems have been developed.	Limited clinical implementation.	Improved discrimination compared with conventional risk models has been reported in selected retrospective cohorts.	Prospective workflow integration, calibration, clinician use, and demonstrable outcome benefit remain insufficiently established.
	Post-discharge remote monitoring [69,70]	Dedicated clinical decision-support and wearable-based monitoring systems have been developed.	Pilot and early clinical implementation.	Earlier identification of postoperative complications and more targeted monitoring have been reported.	Larger prospective studies are required to determine effects on readmissions, clinician workload, false-positive alerts, and patient outcomes.
**Multimodal AI**	Imaging + EHR + genomics integration [73,74,75,76,77,78,79,82]	No broadly established regulatory pathway; most platforms remain investigational.	Predominantly research use.	Incremental predictive performance over individual data sources has been reported in selected studies.	Missing data, modality contribution, interoperability, leakage, transportability, and benefit over simpler models remain insufficiently addressed.
	Transformer/GNN/LLM approaches [49,74,77,82,83]	Regulatory status remains application-specific and generally early.	Predominantly research, educational, or pilot use.	Potential gains in data integration, documentation, image analysis, and knowledge support have been reported.	Hallucination, bias, explainability, computational burden, accountability, and clinical safety remain unresolved.
**System-Level AI**	Clinical decision support/triage [77,80,81,88,89,90]	Selected commercial and regulated systems are available, although status varies substantially by task.	Heterogeneous and institution-specific implementation.	Potential improvements in prioritisation, risk stratification, and workflow efficiency have been reported.	Effects on clinician behaviour, diagnostic error, automation bias, workload, patient safety, and outcomes remain insufficiently demonstrated.
	Medical education/simulation [85,87]	Commercial and institutional platforms are available.	Increasing use in selected educational programmes.	Improvements in selected learning, assessment, and simulation outcomes have been demonstrated.	Durability of learning, transfer to real-world clinical performance, faculty oversight, and cost-effectiveness remain unclear.
	Clinical trial support [91,92]	Commercial NLP, recruitment, and analytics platforms are available.	Limited use in selected trials and research networks.	Potential reductions in administrative workload and improved patient identification have been reported.	Regulatory acceptability, auditability, algorithmic bias, representativeness, and effects on trial quality remain insufficiently established.

Regulatory or commercial status is distinct from actual clinical implementation. Demonstrated benefit refers to reported improvements in workflow, diagnostic or therapeutic decision-making, patient outcomes, or resource utilisation, rather than technical performance alone. The absence of demonstrated clinical benefit does not necessarily indicate ineffectiveness but reflects limited prospective or comparative evidence. Regulatory and commercial status is jurisdiction- and product-specific and should be verified against current official regulatory sources before publication.

## Data Availability

No new data were created or analyzed in this study. Data sharing is not applicable to this article.

## Abbreviations

The following abbreviations are used in this manuscript:

AI	Artificial Intelligence
CAD	Computer-Aided Diagnosis
CAC	Coronary Artery Calcium
CMR	Cardiac Magnetic Resonance
CNN	Convolutional Neural Network
CT	Computed Tomography
cCTA	Coronary Computed Tomography Angiography
CV	Cardiovascular
ECG	Electrocardiogram
EF	Ejection Fraction
EHR	Electronic Health Record
FFR-CT	Fractional Flow Reserve derived from Computed Tomography
GAN	Generative Adversarial Network
GAT	Graph Attention Network
GCN	Graph Convolutional Network
GNN	Graph Neural Network
GRU	Gated Recurrent Unit
LLM	Large Language Model
LSTM	Long Short-Term Memory
MACE	Major Adverse Cardiovascular Events
MRI	Magnetic Resonance Imaging
PET	Positron Emission Tomography
RNN	Recurrent Neural Network
SPECT	Single-Photon Emission Computed Tomography
SMV	Support Vector Machine
VAE	Variational Autoencoder
ViT	Vision Transformer
